# 

**Published:** 1889-07-13

**Authors:** 


### SPECIAL ANNOUNCEMENT.

### MISS FLORENCE NIGHTINGALE.

The next (August) monthly part of The Hospital, and the weekly issue for 27th July, will contain a biographical sketch of Miss Florence Nightingale, tracing the remarkable career of that devoted lady, and showing the effect of her labours at the present time. The article in the August number will be accompanied by a portrait of Miss Nightingale, which has been reproduced at great cost and perfection; and it will at last be possible for every member of the nursing profession to possess the likeness of one who, by a life of self-sacrifice and philanthropic labour, has raised herself to pre-eminence among women. The monthly number will be issued at sixpence as usual, but orders should be sent early.

### NOTICE TO CORRESPONDENTS.

All MS., Letters, Books for Review, and other matters intended for the Editor, should be addressed The Editor, The Lodge, Porchester Square, London, W.

### Notice to Advertisers and Subscribers.

All Advertisements, Orders for Copies of The Hospital, and Business Communications should be addressed to The Publisher, *not to the Editor*, The Hospital, 139-140, Salisbury-court, Fleet-street, E.C.

Vol. V., now ready, 4s., post free. Cases for binding, may be had of the Publisher, at 1s. 6d. each.

### To Residents, Secretaries, and Matrons.

The Editor will be glad to have the Regulations and each Report as issued by Asylums, Hospitals, Convalescent and Nursing Institutions, as well as those of other Charities, and an early copy in duplicate of all Appeals and Festival Notices, etc., addressed to *The Editor, The Lodge, Porchester-square, London, W.* Any superintendent, secretary, matron, or other chief official who regularly sends such information may be registered, on application to the Publisher, to receive free of cost a cover for binding The Hospital in half-yearly volumes.

### Children's Country Holidays Fund.

Answers should be sent to the Secretary of the Fund, 10, Buckingham-street, Strand, W.C.

### Wants.

(1) *Helpers at end of July wanted.*—London ladies or gentlemen who would give some time at the end of July, when most arrangements for sending the children away have to be made, will be warmly welcomed.

(2) *Help for Paddington.*—The council wishes to form a committee to deal with the poorer parts of Paddington, and would like the names of those who know the poor there.

(3) *Wanted Supervisors and Country Homes in Villages.*—The C.C.H.F. wants country residents who will find and supervise country homes from the middle of July till the middle of August; 5s. a week is paid for each child. The villages should be within 50 miles of London. Cottagers who will take the very poorest and make special arrangements to ensure cleanliness are especially needed.

(4) *Received with Thanks.*—From Nurse Pitchford, a P. O. for 2s. 6d.

### Amusements and Relaxation.

### Second Quarterly Word Competition Commenced July 6th, ends September 28th.

Three Prizes of 15s., 10s., 5s., will be given for the largest number of words derived from the words set for dissection.

Proper names, abbreviations, foreign words, words of less than four letters, and repetitions are barred; plurals, and past and present participles of verbs, are allowed. Nuttall's *Standard* dictionary only to be used.

N.B.—Word dissections must be sent in WEEKLY, arranged alphabetically, with correct total affixed.

The word for dissection for this, the *Second* week of the quarter, being

"PORTSMOUTH."

### Results of Thirteenth Week.

| Names. | July 4th. | Totals. | Names. | July 4th. | Totals. |
|---|---|---|---|---|---|
| Patience | 35 | 847 | Pollie | — | 555 |
| Nurse Duty | 32 | 825 | W. G. R. | — | 518 |
| Lightowlers | 34 | 832 | Robe | — | 150 |
| Scrooge | — | 541 | Beryl | — | 391 |
| Reldas | — | 250 | Daffodil | — | 89 |
| Tinie | 36 | 847 | A Schoolgirl | 15 | 308 |
| K. G. | 23 | 757 | Alice R. | — | 63 |
| Broxbourne | 33 | 818 | Belfast | — | 139 |
| Lauriston | 33 | 820 | Gentian | — | 121 |
| Spes | 31 | 784 | Hope | — | 39 |
| Gretta | — | 224 | Louie | — | 139 |
| Francesca | — | 476 | Hamilton | — | 121 |
| Heatherbell | — | 185 | E. M. | — | 120 |
| Sycamore | — | 201 | F. J. D. | — | 112 |
| Arctus | — | 138 | Ladyr | — | 102 |
| Amicus | 33 | 795 | Surgical Nurse | — | 128 |
| Scratcher | — | 197 | L. Pearce | — | 32 |
| Jemima | — | 138 | Contentio | — | 98 |
| Concours | — | 427 | M. W. | — | 74 |
| C. J. S. | — | 134 | Esperance | — | 75 |
| See-Saw | 32 | 735 | H. B. | — | 10 |
| Rosemont | - | 173 | Cowboy Bill | 31 | 31 |
| Jenny Wren | 32 | 555 | Nesta | — | 124 |
| Eric | — | 496 | Sheeny | — | 111 |
| Tiny | 29 | 736 | Montague | — | 79 |
| N'importe Qui | 31 | 671 | Violet | — | 95 |

RESULTS OF FIRST QUARTERLY WORD COMPETITION.

*First Prize*, 15s.—A tie between PATIENCE (Miss C. Calise, Newbury, Berks.), and TINIE (Miss Todd, Newbury.)

*Second Prize*, 10s.—LIGHTOWLERS (Miss C. Chadwick, 98, Huskisson-street, Liverpool.)

*Third Prize*, 5s.—NURSE DUTY (Miss A. Slaughter, Royal Hospital, Putney.)

*Erratum.*—NURSE DUTY.—See eleventh week totals, for 694 read 684.

### Notice to all Correspondents.

N.B.—All letters referring to this page which do not arrive at 139 and 140, Salisbury-court, London, E.C., by the *first post on Thursdays*, and are not addressed PRIZE EDITOR, will in future be disqualified and disregarded

Competitors can enter for *all* quarterly competitions, but no competitor may take more than *two quarterly prizes* during the year.

### Conditions.

I. Every paper sent in must be clearly written, and one side only must be used. All competitors must mark at the head of their papers the number of their competition.

II. Each paper must be signed by the author with his or her real name and address. A *nom de plume* may be added if the writer does not desire to be referred to by us by his real name. In the case of all prize-winners, however, the real name and address will be published.

III. All communications relating to prize competitions should be addressed to "The Prize Editor, THE HOSPITAL, 139 and 140, Salisbury-court, London, E.C." Competitors are requested not to include in letters, etc., to the Prize Editor communications on matters of other business. All letters must be fully prepaid.

IV. The Prize Editor of THE HOSPITAL is the sole judge of the competitions, and his decision is in all cases final and without appeal.

V. The Prize Editor reserves the right to publish any paper sent in, whether it receives a prize or not. He does not hold himself responsible for any MSS. sent, nor can he undertake to return rejected competitions.

### Scraps and Gleanings.

SCARLET FEVER and typhoid are prevalent at Leeds.

THE death is announced of Surgeon-Major O'Donnell.

CHRIST CHURCH, Greenwich, collected £57 for the hospitals.

DEATHS from measles are now reduced to about 33 a week in London.

THE outbreak of diphtheria at St. John's, Newfoundland, is subsiding.

LOUTH HOSPITAL treated 92 in-patients and 753 dispensary patients last year.

DR. GREENE has been elected resident medical officer of Dublin Union Workhouse.

THE collection-boxes for Richmond Hospital have yielded £176 in the last six months.

THE Hamilton Association keeps 27 men nurses at work. It is not yet self-supporting.

OVER 300 students attend Signora Cattani's lectures on pathology at Bologna University.

MADAME BLEIN is the doctor in charge of the Victoria Jubilee Hospital and Dispensary.

THE *Liverpool Post* of July 3 contained a long and interesting article on "The Hospital Question."

MISS ANNIE HARDING, of Long Ashton, has secured the medical scholarship of £75 per annum.

GLOUCESTER INFIRMARY AND EYE INSTITUTION has issued a special appeal for further subscribers.

SIR MORELL MACKENZIE received a magnificent presentation from the dramatic profession last Saturday.

MR. HEATH, Mr. Hulke, and Mr. Howse have been elected to the council of the Royal College of Surgeons.

A FLOWER SERVICE was held at Gunnersbury last Sunday, and the flowers sent to the West London Hospital.

MISS B. B. BOSE, a Bengalee Christian, has passed the first L. M. S. Examination of the Calcutta Medical College.

A CONCERT was given on Tuesday last in aid of the London Throat Hospital. The Lotus Glee Club sang excellently.

THE Duke of Newcastle lately presided over the annual meeting of the Society for the Protection of Women and Children.

SHRIVENHAM collected £10 on Hospital Sunday, but ended the day by a great deal of drinking and blasphemous language.

BARNSLEY BECKETT HOSPITAL treated 281 in-patients, and over 2,000 out-patients last year, at an average cost of £1 11s. 2d.

HAMPSTEAD PARISH CHURCH collections on Hospital Sunday amounted to £58. The Rev. S. B. Burnaby preached in the morning.

ONE of the recent visitors to the Paris Exhibition is M. Harduin, aged 102 years. He intends, it is said, to ascend the Eiffel Tower.

LADY PEEK distributed the prizes at the National Orphan Home, Ham, last week. Mr. Brandram kindly gave some humorous recitations.

NEW BRUNSWICK has a lazaretto. When it was established 20 years ago there were 400 lepers, now segregation has reduced them to nine.

THE Berlin Society of Domestic Hygiene, of which the Empress Frederick is patroness, boards out about 1,500 children yearly in the country.

GENERAL practitioners in South London hear with regret that a Mr. Macgillicuddy, of South Kensington, proposes to start a temperance hospital in that locality.

COLONEL BRISTOW, for twenty years secretary to the Miller Hospital, was presented last week with silver plate to the value of £200, by subscribers to the institution.

THE amount of the collections on Hospital Sunday at the Parish Church, Bromley, was £71, which is divided between the Metropolitan Hospital Fund and the Bromley Cottage Hospital.

THE late Dr. Fairbrother, of Berkeley-square, Bristol, has left £1,000 to the Royal Infirmary, Bristol, to endow a bed to his memory and £500 for a child's cot in memory of the late Mrs. Fairbrother.

A NUMBER of ladies at Oxford will shortly join the St. John Ambulance Corps. Their drill will be on a different evening to the men's. Eighteen ladies have just received certificates and are qualified to render first aid.

BY command of the Queen, Sir Henry Ponsonby, Keeper of the Privy Purse, has informed the Lord Mayor that it will give Her Majesty much pleasure to send a donation of £200 to the Patriotic Volunteer Fund.

BY the kind permission of the Countess of Dudley a sale of Scottish home industries was held last week at Dudley House, Park-lane, the object being to promote an interest in London in the home produce of Scotland.

THE Lord Mayor has received a donation for the Hospital Sunday Fund of £367 16s., representing a collection among members of the Stock Exchange, which has been undertaken by Mr. A. L. Cohen and Mr. Kennedy.

THE Duke of Edinburgh, who will this week terminate his course of treatment at Kissingen, has, says the *British Medical Journal*, so much improved that he now considers himself in the enjoyment of his usual health and strength.

LORD KINNAIRD presided over the festival dinner in aid of the London Lock Hospital. During the past five years out of 3,000 patients treated 800 have been rescued. About £2,000 has been paid or promised in connection with the dinner.

PRINCE GEORGE OF WALES has been compelled to change the date for laying the foundation-stone of the new branch hospital of the Seamen's Hospital Society at the Albert and Victoria Docks from Thursday, the 18th, to Monday, the 15th inst.

OXFORD LOCAL BOARD reports a case of rabies in a dog belonging to a lady. The animal had been destroyed, but it had bitten several dogs which could not be identified. An order was issued requiring all dogs to be muzzled when at large, and to provide for dealing with those suspected of or affected with rabies.

